# Management of Marginal Zone Lymphoma: A Canadian Perspective

**DOI:** 10.3390/curroncol30020135

**Published:** 2023-02-01

**Authors:** Anthea Peters, Mary-Margaret Keating, Anna Nikonova, Sarah Doucette, Anca Prica

**Affiliations:** 1 Department of Oncology, Cross Cancer Institute, University of Alberta, Edmonton, AB T6G 1Z2, Canada; 2 Division of Hematology, Queen Elizabeth II Health Sciences Centre, Dalhousie University, Halifax, NS B3H 2Y9, Canada; 3 Division of Hematology, McGill University Health Centre, Montreal, QC H4A 3J1, Canada; 4 Impact Medicom Inc., Toronto, ON M6S 3K2, Canada; 5 Division of Medical Oncology and Hematology, Princess Margaret Cancer Centre, Toronto, ON M5G 2C1, Canada

**Keywords:** marginal zone lymphoma, mucosa-associated lymphoid tissue lymphoma, chemoimmunotherapy, anti-CD20 monoclonal antibody, Bruton’s tyrosine kinase inhibitor

## Abstract

Marginal zone lymphomas (MZL) are a rare, heterogenous group of lymphomas, accounting for 5–17% of indolent non-Hodgkin lymphomas in the western world. They can be further divided into three subtypes: extranodal MZL, splenic MZL, and nodal MZL. These subtypes differ in clinical presentation and behavior, which influences how they are managed. There is currently no standard of care for the treatment of MZL, owing to the difficulty in conducting phase 3 randomized trials in MZL, and the fact that there are limited data on the efficacy of therapy in individual subtypes. Treatment practices are thus largely borrowed from other indolent lymphomas and are based on patient and disease characteristics, as well as access to therapy. This review summarizes the Canadian treatment landscape for MZL and how these therapies may be sequenced in practice.

## 1. Introduction

Marginal zone lymphomas (MZL) are a heterogenous group of indolent non-Hodgkin lymphomas (iNHL) that stem from memory B lymphocytes typically located in the marginal zone of secondary lymphoid organs. There are three subtypes of MZL that share similar histologic and immunophenotypic features but differ in presentation and clinical behavior. These include extranodal MZL of mucosa-associated lymphoid tissue (EMZL or MALT lymphoma), splenic MZL (SMZL), and nodal MZL (NMZL). Marginal zone lymphomas account for 5–17% of iNHLs in the western world [1]. The median age at diagnosis across all MZL subtypes is 60–70 years and the incidence increases with age [2,3,4,5]. Marginal zone lymphoma is frequently indolent in nature, with many patients surviving beyond 10 years from diagnosis. However, in the approximately 20% of MZL patients who relapse or progress within 2 years, median overall survival (OS) is only 3–5 years [6,7]. 

The optimal treatment for MZL is not clear owing to the limited representation of patients with MZL in phase 3 trials for iNHL. In addition, phase 2 studies evaluating therapeutic efficacy in MZL generally lack a control arm and pool patients of different subtypes that have heterogeneous clinical behavior. As MZL lacks specific immunophenotypic markers, it may also be difficult to distinguish from other lymphoproliferative disorders with similar morphology and clinical presentation, which can challenge trial interpretation and patient management. Thus, in many cases, treatment strategies for MZL are borrowed from other iNHLs such as follicular lymphoma (FL). It is plausible, however, that MZL may respond differently to therapy than other iNHLs given that the clinical presentation, genetics, and natural history of MZL is generally different [8]. Therefore, further study is needed to understand the best treatment for MZL patients.

Treatment decisions in MZL are also largely driven by drug access. New drugs must first be authorized for sale by Health Canada, a governing body that reviews the evidence submitted by the manufacturer on the safety, efficacy, and quality of a drug. Once Health Canada has issued a notice of compliance, the drug may be accessible through private insurance plans or manufacturer-led compassionate access programs. Public funding decisions for drugs are made at the provincial level and aided by recommendations from health technology assessment bodies and price negotiations from the pan-Canadian Pharmaceutical Alliance. However, this does not guarantee access uniformity across the country. This review discusses treatment options for MZL and how these therapies are utilized in Canada.

## 2. Clinical Characteristics of MZL Subtypes

The distinction of all three MZL subtypes as separate entities was only formally recognized in 2001 with the World Health Organization’s classification for hematologic and lymphoma tissues [9]. It should be noted that MZL has historically been difficult to differentiate from other CD5-/CD10-lymphoproliferative disorders, particularly lymphoplasmacytic lymphoma/Waldenstrom Macroglobulinemia, which can have similar morphologic findings and disease presentation [10]. Consultation with an experienced hematopathologist and use of MYD88 mutational testing can help distinguish these two entities [11]. 

Extranodal MALT lymphoma is the best characterized and most common MZL subtype, accounting for 50–70% of cases [1]. It also has the best prognosis among subtypes, with a 5-year relative survival rate of 94% [3]. Extranodal MZL can manifest in any tissue, most commonly the stomach (30%), eye/adnexa (12%), skin (10%), lung (9%), and salivary gland (7%) [3]. Patients typically present with symptoms caused by lymphoma involvement at the affected tissue site. Most MALT lymphomas remain localized for a prolonged time, although multi-focal single organ involvement or disseminated disease occur in up to 25% of cases (most often with non-gastric sites) [12].

Splenic MZL is the second most common subtype, accounting for approximately 20% of cases and having a 5-year relative survival rate of 85% [1,3]. It typically presents with splenomegaly and infiltration to the bone marrow, and often the blood [13], with minimal lymphadenopathy except for the splenic hilum. Approximately one third of patients have no symptoms at diagnosis [5]. Monoclonal protein is observed in about one third of patients and is predominantly immunoglobulin M (about 60%) or immunoglobulin G (about 30%) [14]. Paraprotein-related conditions are present in 15–20% of patients at diagnosis [5,15]. These may include autoimmune hemolytic anemia, immune thrombocytopenia, cold agglutinin disease, acquired von Willebrand disease, anti-phospholipid antibody syndromes, C1 esterase inhibitor deficiency, immunoglobulin light chain amyloidosis, and anti-myelin associated glycoprotein neuropathy [5,15,16,17,18].

Nodal MZL is the least common (10% of cases) of the MZL subtypes, with a 5-year relative survival rate of 83% [3]. Histologic and immunophenotypic features of NMZL are similar to those of EMZL and SMZL, but NMZL is characterized by disseminated lymphadenopathy [19]. Other clinical features at diagnosis may include bone marrow involvement (about one third of patients), B symptoms (up to 20%), anemia and/or thrombocytopenia (10–25%) [20]. Similar to SMZL, monoclonal protein may be present in 10–30% at diagnosis [20], which can lead to paraprotein-related conditions.

## 3. Indications for Treatment

As MZL is generally indolent in nature, active surveillance (watch and wait) is the accepted management approach for asymptomatic patients regardless of stage, similar to other iNHLs. This is supported by several retrospective studies that demonstrated excellent survival rates for patients on an active surveillance protocol [21,22,23,24]. An exception would be asymptomatic patients with stage I/II gastric MALT lymphoma who are positive for Heliobacter pylori (*H. pylori*), where antibiotic treatment would be indicated (discussed below). Indications for treatment of MZL include symptomatic splenomegaly or lymphadenopathy, presence of B symptoms (weight loss >10% in 6 months, fever >38 °C, drenching night sweats), cytopenia (hemoglobin <10 g/dL, platelets <80,000/µL, neutrophils <1000/µL), and/or bulky disease [19]. Once an indication for treatment is determined, therapy is selected based on patient characteristics, such as age, comorbidities, and treatment goals, and tumor features such as MZL subtype and stage.

## 4. First-Line Treatment Options

### 4.1. Pathogen-Directed Therapy

Development of MZL, and particularly EMZL, is often linked to chronic antigenic stimulation caused by persistent infections, treatment of which can lead to lymphoma regression [3]. The most studied infectious agent associated with increased risk of MZL is *H. pylori*, which is detected in approximately two thirds of gastric MALT lymphomas, although the rates of *H. pylori*-positive EMZL have been declining in some regions [25,26]. Treatment of *H. pylori* with antibiotics and proton pump inhibitors has demonstrated a lymphoma regression rate of over 75% in clinical studies of patients with limited-stage gastric EMZL; however, it requires endoscopic surveillance to ensure eradication [27]. Eradication therapy may also benefit a small subset of *H. pylori*-negative patients, which may be explained by false-negative test results, infection with another Helicobacter species, or a direct anti-tumor effect of the antibiotics used [28,29]. Presence of chromosomal translocation t(11;18) confers a low probability of response to antibiotic H. Pylori eradication treatment and is not recommended in these patients [30]; however, testing for t(11;18) is not routinely performed in all provinces. 

*Chlamydia psittaci* (*C. psittaci*) has been linked to EMZL of the eye/adnexa and doxycycline therapy has demonstrated efficacy in these patients in small prospective clinical trials [31]. However, *C. psittaci* is not routinely tested in Canada given the geographic variability in incidence of *C. psittaci*-positive ocular adnexal MALT lymphoma (0–87%) [32,33,34,35,36], and the lack of Canadian data. The causal association between other bacterial species and EMZL at specific sites continue to be explored. Notably, *Borrelia burgdorferi* and *Campylobacter jejuni* infections have been reported in cases of EMZL originating in the skin and small intestine, respectively, with case reports demonstrating responses to antibiotic therapy in these patients [37,38,39]. Development of EMZL at specific sites has also been linked to chronic antigen stimulation caused by autoimmune conditions such as Sjögren syndrome (salivary gland) and Hashimoto thyroiditis (thyroid) [40,41,42,43,44]; however, the co-occurrence of one of these conditions with MZL does not influence MZL treatment. 

An association between chronic hepatitis C virus (HCV) infection and B-cell NHL has been reported in several epidemiological studies with up to a 3-fold increase in risk of B-cell NHL in HCV-infected individuals, although this risk varies by the prevalence of HCV infection in different populations [45]. Among the B-cell NHL subtypes, diffuse large B-cell lymphoma, lymphoplasmacytic lymphoma, and MZL (most often SMZL) show the highest prevalence of concurrent HCV infection [45]. There is real-world evidence demonstrating lymphoma regression after antiviral therapy in patients with HCV-positive MZL, thus antiviral medication should be given in HCV-positive patients who do not require immediate disease control [45,46,47].

### 4.2. Local Therapy

#### 4.2.1. Involved-Site Radiation Therapy

Involved-site radiation therapy (ISRT) is the standard first-line treatment for localized EMZL, except in patients with *H. pylori*-positive, t(11;18) negative gastric MALT lymphoma, where a trial of *H. Pylori* eradication can be first considered. This is based on excellent response rates and a median progression-free survival (PFS) over 10 years in this patient population [48,49,50]. Dosing between 20–30 Gy is generally recommended; however lower doses may be beneficial in certain situations such as treatment for radiosensitive organs (e.g., eyes) and for palliative symptom control [51]. Emerging retrospective data on low-dose ISRT, consisting of two consecutive 2 Gy fractions, have demonstrated frequent and sustained responses in patients with ocular adnexal and other MALT lymphomas without any significant acute or chronic adverse events [52,53].

#### 4.2.2. Splenectomy

Splenectomy remains a common first-line treatment option for SMZL at several Canadian centers and can help facilitate diagnosis in patients with symptomatic splenomegaly with no other sites of disease. It is associated with response rates over 85% and 5-year OS rates of 60–85% in retrospective studies [54,55,56,57]. As splenectomy results in complete resolution of splenomegaly-related symptoms and improvement of cytopenia while bone marrow infiltration and lymphocytosis persist, it is best used in patients with minimal bone marrow involvement. Major bleeding may occur in up to 20% of splenectomized patients, with spleen weight being a significant independent risk factor for this perioperative complication [58]. Splenectomy is also associated with long-term risks of fatal infections caused by encapsulated bacteria (reported in approximately 5% of patients), and therefore vaccination against encapsulated bacteria is required at least 2 weeks before surgery [55,57,59]. Patient preference, age, comorbidities, disease bulk, and dissemination must be considered when deciding between splenectomy or other first-line therapy options.

### 4.3. Systemic Therapy

#### 4.3.1. Single-Agent Rituximab

Rituximab, an anti-CD20 monoclonal antibody, is typically used in combination with chemotherapy for the treatment of symptomatic advanced-stage EMZL and NMZL; however, it may be given as monotherapy in select frail patients who are deemed unable to tolerate chemotherapy. This is based on results from a phase 2 study of 35 patients with treatment-naïve or relapsed EMZL, which showed moderate response rates with four weekly doses of rituximab 375 mg/m^2^ (overall response rate [ORR]: 73%; complete response [CR]: 43%), although responses were short (median time to treatment failure: 14.2 months) [7]. Subsequent small retrospective studies of patients with EMZL and NMZL have reported similar findings [60,61]. 

Single-agent rituximab has a more established role in the treatment of SMZL, as first-line treatment with rituximab has been found to be well-tolerated and can achieve ORRs of approximately 90% in retrospective studies [56,62,63]. In one of these studies, evaluating 58 patients receiving rituximab (375 mg/m^2^ for 6 weeks followed by maintenance therapy for responders every 2 months for 1–2 years) and 27 patients treated with splenectomy, OS was found to be similar in patients treated with each modality (5-year OS: 92% and 77%, respectively; *p* = 0.09) [56]. Infusion-related reactions occurred in most patients receiving rituximab and were easily managed with supportive care. Other reported toxicities for rituximab included renal dysfunction and grade 3 thrombocytopenia, both of which led to treatment discontinuation, as well as three patients experiencing grade 2 neutropenia. A follow-up to this series that included 108 patients found that among rituximab responders, patients receiving maintenance therapy had significantly longer 5-year free-from progression rates compared with patients who did not receive maintenance (79% vs. 52%, *p* = 0.0006) [64]. While data to support single-agent rituximab for the first-line treatment of SMZL remain limited and based only on retrospective analyses, some provinces prefer its use over splenectomy due to its perceived favorable safety profile.

#### 4.3.2. Chemoimmunotherapy

Chemoimmunotherapy is the preferred first-line treatment for patients with symptomatic, advanced-stage MZL (except SMZL, see above). Bendamustine-rituximab (BR), cyclophosphamide-doxorubicin-vincristine-prednisone-rituximab (CHOP-R), and cyclophosphamide-vincristine-prednisone-rituximab (CVP-R) have been evaluated in two phase 3 randomized studies in patients with iNHL or mantle cell lymphoma (StiL NHL1 and BRIGHT), although MZL represented less than 15% of the population [65,66]. Both studies met their primary endpoint, with the StiL NHL1 study showing noninferior PFS for BR compared to R-CHOP (median PFS 69.5 vs. 31.2 months, respectively; HR 0.58; *p* < 0.0001 for non-inferiority) and the BRIGHT study showing noninferior CR for BR compared to R-CHOP or R-CVP (31% vs. 25%, respectively; *p* = 0.0225 for non-inferiority) [65,67]. Interestingly, an analysis by histological subtype showed that MZL was the only group to not demonstrate a statistically significant improvement in PFS for BR versus CHOP-R in the StiL NHL1 study [67]. Though the subgroup was not adequately powered to measure this, it may reflect the overall lower chemosensitivity of MZL. Given that BR was better tolerated than R-CVP/R-CHOP in both trials, with lower rates of high-grade cytopenia and infection reported, it is generally the preferred chemoimmunotherapy in all MZL subtypes [19]. 

Additional phase 2 studies support the use of BR in patients with specific subtypes of MZL. The phase 2 MALT2008-01 reported a CR rate (including unconfirmed CR) of 75% in patients with EMZL after 3 cycles of BR and a 7-year event free survival rate of 88% [68]. Responses did not appear to differ by primary site or t(11;18) status. The BRISMA/IELSG36 study also reported an ORR of 91% and 3-year OS of 96% in patients with previously untreated SMZL or in patients who had relapsed after splenectomy [69]. 

Chlorambucil and rituximab has also demonstrated efficacy for the first-line treatment of advanced symptomatic EMZL [70]; however, this regimen is infrequently used in Canada. Similarly, excellent response rates and prolonged remissions have been reported for fludarabine and rituximab in phase 2 clinical trials; [71,72] however, it is not used frequently in Canada due to the availability of more efficacious, less toxic options, such as BR (Table 1). 

#### 4.3.3. Maintenance Rituximab

Rituximab maintenance is routinely used in Canada following induction with BR based on results from the MZL analysis of the phase 3 StiL NHL7-2008 MAINTAIN trial (104 randomized patients), which showed a superior PFS for rituximab maintenance over observation (median PFS not reached vs. 92.2 months, HR 0.35, 95% CI 0.17–0.76; *p* = 0.008) [73]. However, given that a statistically significant OS benefit was not observed (HR 0.52, 95% CI 0.20–1.39), the option of maintenance therapy should be discussed with the patient and should consider the benefit of prolonged PFS as well as the potential risks associated with the B-cell depletion caused by rituximab. These include increased risk of infection and poor immune response following vaccination, which has become a greater concern in light of the severe acute respiratory syndrome coronavirus 2 (SARS-CoV-2) pandemic [74,75,76]. A paucity of data also remains on the role of maintenance therapy in patients with advanced MALT lymphoma as these patients were not included in the MAINTAIN trial. 

#### 4.3.4. Lenalidomide-Rituximab

Lenalidomide-rituximab (R2) has demonstrated ORRs of 80–90% and median PFS of 54 months in phase 2 trials of patients with treatment-naïve MZL [77,78]. Lenalidomide-rituximab is not frequently used in Canada for MZL due to the lack of funding by provincial health agencies. 

**Table 1:** Phase 2/3 prospective trials for first-line treatment of advanced/symptomatic MZL.

Study Name/Regimen	Phase	Population	CR (%)	PFS		OS	
				Median/Landmark	HR (95% CI)	Median/Landmark	HR (95% CI)
StiL NHL1 [66,67] BR vs. CHOP-R	3	Advanced iNHL or MCL N_(ITT)_ = 514 N_(MZL)_ = 67	ITT: 40 vs. 30 *p* = 0.021 MZL: Not reported	Median_(ITT)_: 69.5 vs. 31.2 months Median_(MZL)_: 57.2 vs. 47.2 months	ITT: 0.58 (0.44–0.74) *p* < 0.0001 MZL: 0.70 (0.34–1.43) *p =* 0.3249	10-year_(ITT)_: 71% vs. 66% 10-year_(MZL)_: Not reported	ITT: 0.82 (0.58–1.15) *p* = 0.249 MZL: Not reported
BRIGHT [65,79] BR vs. CHOP-R or CVP-R	3	Advanced iNHL or MCL N_(ITT)_ = 447 N_(MZL)_ = 46	ITT: 31 vs. 25 *p* = 0.0225 * MZL: 20 vs. 24	5-year_(ITT)_: 66% vs. 56% Median_(MZL)_: Not reported	ITT: 0.61 (0.45–0.85) *p =* 0.0025 MZL: Not reported	5-year_(ITT)_: 82% vs. 85% 5-year_(MZL)_: Not reported	ITT: 1.15 (0.72–1.84) *p* = 0.5461 MZL: Not reported
MAINTAIN [73] R-maint vs. Obs	3	Advanced NMZL/SMZL after response to BR N = 104	N/A	Median: NR vs. 92.2 months	0.35 (0.17–0.76) *p =* 0.008	6-years: 92% vs. 86%	0.52 (0.20–1.39)
IELSG-19 [70] Clb-R vs. Clb vs. R	3	EMZL, no prior systemic therapy N = 401	79 vs. 63 vs. 56	Median: NR vs. 8.3 years vs. 6.9 years	Clb-R vs. Clb: 0.62 (0.42 to 0.93) *p =* 0.0119	5-year: 90% vs. 89% vs. 92%	Clb-R vs. Clb: 1.24 (0.69–2.23) *p* = 0.464
Salar et al., 2009 [71] FR	2	EMZL-MALT N = 22	90	2-year: 88%	N/A	2-year: 100%	N/A
Brown et al., 2009 [72] FR	2	Advanced MZL N = 26	54	3-year: 80%	N/A	3-year: 87%	N/A
AGMT MALT-2 [78] R2	2	EMZL-MALT N = 46	54	27-month: 91%	N/A	Not reported	N/A
Fowler et al., 2014 [77] R2	2	Advanced FL, MZL, SLL N_(ITT)_ = 110 N_(MZL)_ = 30	ITT: 63 MZL: 67	Median_(ITT)_: 53.8 months Median_(MZL)_: 53.8 months	N/A	3-year_(ITT)_: 96% 3-year_(MZL)_: 100%	N/A
MALT 2008-01 [68,80] BR	2	EMZL-MALT N = 57	98	7-year: 93%	N/A	7-year: 96%	N/A
BRISMA/ IELSG36 [69] BR	2	SMZL N = 56	73	3-year: 90%	N/A	3-year: 96%	N/A

* *p*-value for non-inferiority. BR, bendamustine-rituximab; CHOP-R, cyclophosphamide-doxorubicin-vincristine-prednisone-rituximab; CI, confidence interval; Clb-R, chlorambucil-rituximab; CR, complete response; CVP-R, cyclophosphamide-vincristine-prednisone-rituximab; EMZL, extranodal marginal zone lymphoma; FL, follicular lymphoma; FR, fludarabine-rituximab; HR, hazard ratio; iNHL, indolent non-Hodgkin lymphoma; ITT, intention-to-treat; maint, maintenance; MALT, mucosa-associated lymphoid tissue; MCL, mantle cell lymphoma; MZL, marginal zone lymphoma; N/A, not applicable; NMZL, nodal marginal zone lymphoma; NR, not reached; Obs, observation; OS, overall survival; PFS, progression-free survival; R, rituximab; R2, lenalidomide-rituximab; SLL, small lymphocytic lymphoma; SMZL, splenic marginal zone lymphoma.

## 5. Treatment of Relapsed/Refractory MZL

There is no standard of care for treatment of MZL in the relapsed setting. In Canada, treatment for relapsed, symptomatic, advanced-stage MZL may include anti-CD20-based chemoimmunotherapy, Bruton’s tyrosine kinase inhibitor (BTKi) therapy, or R2. BTKi therapy is currently only accessible in Canada through compassionate access programs, while R2 is not widely available across provinces and may be accessible through private drug coverage. Several class 1 PI3K inhibitors have also shown efficacy in MZL (Table 2); however, adverse events including severe diarrhea/colitis, hypertension, hyperglycemia, and signals for increased death have restricted their use in the United States and none of these agents are approved or funded in Canada [81,82,83,84,85,86].

Treatment selection is based on types of prior therapies, duration of response to those therapies, and patient fitness. If the patient had a prolonged remission after completing first-line chemoimmunotherapy (>2–4 years, depending on treater), treatment may be repeated at relapse. Early relapse (within 24 months of starting therapy) occurs in about 20% of patients and a biopsy should be performed in these patients to rule out large cell transformation, which requires more aggressive treatment [6,87]. 

For patients who are refractory to rituximab, defined as relapsing within 6 months of completing rituximab therapy, the anti-CD20 antibody obinutuzumab has demonstrated efficacy in combination with bendamustine for patients with iNHL [88,89] (Table 2). Although the Health Canada indication for obinutuzumab is specific to FL and chronic lymphocytic leukemia, it is reimbursed in combination with chemotherapy for the treatment of rituximab-refractory MZL in most provinces.

Bruton’s tyrosine kinase inhibitors are a consideration for patients with relapsed MZL based on their ease of administration, good tolerability, and durable responses in this setting. A phase 2 study in 63 patients with relapsed/refractory MZL reported an ORR of 58% (CR 10%) and median PFS of 15 months after treatment with the first-generation BTKi ibrutinib [90] (Table 2). Zanubrutinib, a next generation BTKi with higher selectivity for BTK, was investigated in the phase 2 MAGNOLIA trial where it achieved an ORR of 68% (CR 26%) in 68 patients with relapsed/refractory MZL [91]. A recent updated analysis of MAGNOLIA, at a median follow-up of 28 months, reported a 24-month PFS rate of 71% (assessed by an independent review committee using positron emission tomography and/or computed tomography) [92]. Zanubrutinib is approved by Health Canada for the treatment of patients with MZL who have received at least one prior anti-CD20-based therapy, making it the first agent with approval specifically for MZL. Zanubrutinib in combination with rituximab is also being evaluated against R2 in an ongoing phase 3 trial in patients with relapsed/refractory MZL (NCT05100862). While both ibrutinib and zanubrutinib are available through compassionate programs in Canada, zanubrutinib is now preferred based on its improved tolerability compared to ibrutinib as demonstrated in direct comparison trials in other B-cell malignancies [93,94]. Of particular importance is the decrease in atrial fibrillation and cardiac adverse events with zanubrutinib.

Lenalidomide-rituximab has also been studied in relapsed/refractory MZL. In the phase 3 AUGMENT trial in patients with relapsed/refractory FL and MZL, the median PFS in patients treated with R2 was 39.4 months versus 14.1 months for placebo plus rituximab [95] (Table 2). In the subgroup analysis of patients with MZL (n = 63), no difference in PFS or OS was detected between arms; however, the small number of patients and imbalances in baseline characteristics make interpretation of these results challenging. Given the data for R2 for treatment-naïve MZL [77,96], and the data for the overall population in the AUGMENT trial, R2 is reasonable to consider in patients with relapsed MZL.

**Table 2:** Phase 2/3 prospective trials for treatment of relapsed/refractory advanced/symptomatic MZL.

Study Name/ Regimen	Phase	Population	CR (%)	PFS		OS	
				Median/Landmark	HR (95% CI)	Median/Landmark	HR (95% CI)
PCYC-1121 [90] Ibrutinib	2	R/R MZL after SAR or CIT N = 63	10	Median: 15.7 months	N/A	33-month: 72%	N/A
MAGNOLIA [91,92] Zanubrutinib	2	R/R MZL after ≥1 R-based therapy N = 68	26	24-month: 71%	N/A	24-month: 86%	N/A
AUGMENT [95] R2 vs. placebo-R	3	R/R FL or MZL N_(ITT)_ = 358 N_(MZL)_ = 63	ITT: 34 vs. 18 MZL: 29 vs. 13	Median_(ITT)_: 39.4 vs. 14.1 months Median_(MZL)_: 20.2 vs. 25.2 months	ITT: 0.46 (0.34–0.62) *p* < 0.001 MZL: 1.00 (0.47–2.13)	2-year_(ITT)_: 93% vs. 87% 2-year_(MZL)_: 82% vs. 94%	ITT: 0.61 (0.33–1.13) MZL: 2.89 (0.56–14.92)
GADOLIN [89] Bendamustine-obinutuzumab vs. bendamustine	3	Rituximab- refractory iNHL N_(ITT)_ = 396 N_(MZL)_ = 46	ITT: 17 vs. 17 MZL: Not reported	Median_(ITT)_: NR vs. 14.9 months Median_(MZL)_: Not reported	ITT: 0.55 (0.40–0.74) *p =* 0.0001 MZL: 0.94 (0.46–1.90)	Median_(ITT)_: NR vs. NR Median_(MZL)_: Not reported	ITT: 0.82 (0.52–1.30) *p =* 0.40 MZL: Not reported
Gopal et al., 2014 [83] Idelalisib	2	iNHL refractory to R + alkylating agent N_(ITT)_ = 125 N_(MZL)_ = 15	ITT: 6 MZL: Not reported	Median_(ITT)_: 11 months Median_(MZL)_: Not reported	N/A	Median_(ITT)_: 20.3 months Median_(MZL)_: Not reported	N/A
DYNAMO [84] Duvelisib	2	iNHL refractory to R + chemotherapy N_(ITT)_ = 129 N_(MZL)_ = 18	ITT: 2 MZL: 6	Median_(ITT)_: 9.5 months Median_(MZL)_: not reported	N/A	Median_(ITT)_: 28.9 months Median_(MZL)_: not reported	N/A
CHRONOS-1 [81] Copanlisib	2	R/R iNHL, ≥2 prior therapies N_(ITT)_ = 142 N_(MZL)_ = 23	ITT: 17 MZL: 13	Median_(ITT)_: 12.5 months Median_(MZL)_: not reported	N/A	Median_(ITT)_: 42.6 months Median_(MZL)_: not reported	N/A
CHRONOS-3 [86] Copanlisib-R vs. placebo-R	3	iNHL relapsed after anti-CD20 therapy N_(ITT)_ = 458 N_(MZL)_ = 95	ITT: 34 vs. 15 MZL: 39 vs. 10	Median_(ITT)_: 21.5 vs. 13.8 months Median_(MZL)_: 22.1 vs. 11.5 months	ITT: 0.52 (0.39–0.69) *p* < 0.0001 MZL: 0.48 (0.25–0.92) *p =* 0.012	36-month_(ITT)_: 83% vs. 81% 36-month_(MZL)_: Not reported	ITT: 1.07 (0.63–1.82) MZL: not reported
UNITY-NHL [97] Umbralisib	2b	R/R iNHL after ≥1 anti-CD20 therapy N_(ITT)_ = 208 N_(MZL)_ = 69	ITT: 9 MZL: 16	Median_(ITT)_: Not reported Median_(MZL)_: NR	N/A	Not reported	N/A

CI, confidence interval; CIT, chemoimmunotherapy; CR, complete response; FL, follicular lymphoma; HR, hazard ratio; iNHL, indolent non-Hodgkin lymphoma; ITT, intention-to-treat; MZL, marginal zone lymphoma; N/A, not applicable; NR, not reached; OS, overall survival; PFS, progression-free survival; R, rituximab; R, rituximab; R/R, relapsed/refractory; R2, lenalidomide-rituximab; SAR, single-agent rituximab.

## 6. Summary and Canadian Perspective

**Figure 1:**
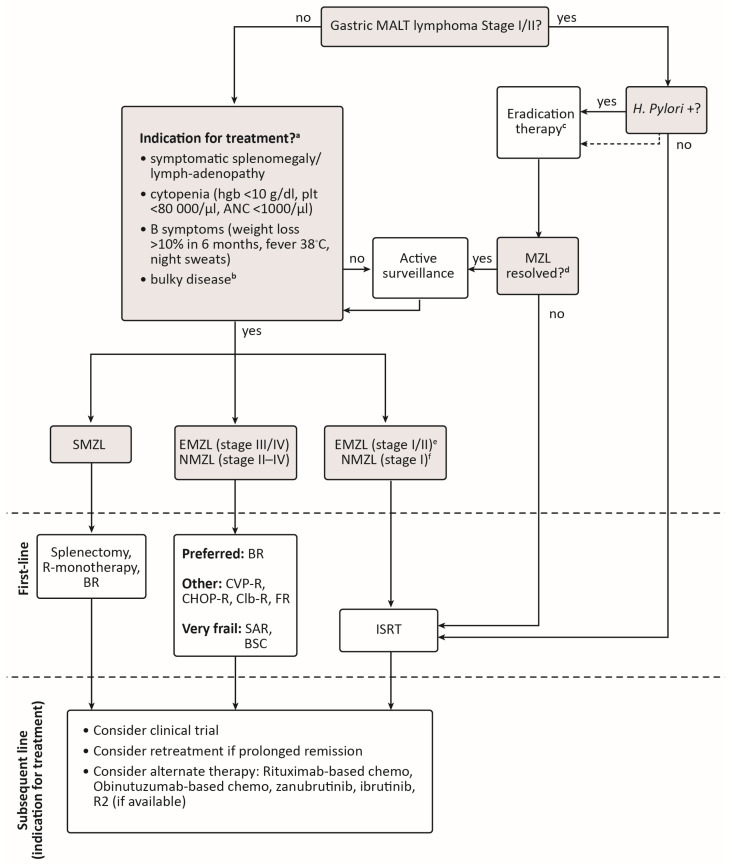
Suggested algorithm for the treatment of marginal zone lymphoma in Canada. ^a^ If patient is positive for Hepatitis C infection, treat first with antiviral therapy if immediate disease control is not needed. ^b^ Bulky disease is not clearly defined for MZL and may be considered as >7 cm as per GELF (Groupe d’Etude des Lymphomes Folliculaires) criteria for follicular lymphoma [104] or >10 cm, depending on individual practice. ^c^ *H. pylori* eradication therapy should be confirmed ≥4 weeks after treatment. If initial eradication therapy is unsuccessful, up to two additional trials of *H. pylori* eradication can be considered. ^d^ If *H. Pylori* eradication is confirmed, then serial scopes with biopsies should be performed every 3 months until a complete lymphoma response is reached. ^e^ Testing for *C. psittaci* in ocular adnexal MALT lymphoma is not routinely performed in Canada but may be considered. In patients who are *C. psittaci*-positive and do not require immediate disease control treatment with doxycycline may be considered. ^f^ Stage I NMZL is extremely rare, but ISRT may be considered in these patients based on extrapolation from studies in follicular lymphoma suggesting a chance for a functional cure [105,106]. ANC, absolute neutrophil count; BR, bendamustine-rituximab; Bruton’s tyrosine kinase; BSC, best supportive care; CHOP-R, cyclophosphamide-doxorubicin-vincristine-prednisone-rituximab; Clb-R, chlorambucil-rituximab; CVP-R, cyclophosphamide-vincristine-prednisone-rituximab; EMZL, extranodal marginal zone lymphoma; FR, fludarabine-rituximab; hgb, hemoglobin; ISRT, involved-site radiation therapy; MALT, mucosa-associated lymphoid tissue; NMZL, nodal marginal zone lymphoma; plt, platelets; R2, rituximab-lenalidomide; SAR, single-agent rituximab; SMZL, splenic marginal zone lymphoma.

The Canadian landscape for first-line treatment of MZL differs slightly between subtypes (Figure 1). Radiation therapy remains the most common treatment for symptomatic limited-stage EMZL and NMZL except for patients with *H. pylori*-positive, t(11;18) negative gastric MALT lymphoma who would generally receive antibiotics and proton pump inhibitors as first-line therapy. Symptomatic SMZL, which typically presents in advanced stages, may be treated with splenectomy, single-agent rituximab, or BR in the first-line setting depending on patient preference, fitness, and extent of disease. Bendamustine-rituximab is the most common chemoimmunotherapy used for first-line treatment of advanced, symptomatic EMZL and NMZL as it induces long remissions in the majority of patients. Efficacy of BR is similar to CHOP-R and CVP-R, but BR is associated with less hematologic toxicity. For older, frail patients, who are deemed unlikely to tolerate BR, rituximab monotherapy can be considered. 

Rituximab maintenance is frequently given following single-agent rituximab or BR as it may deepen responses and prolong remission. However, since rituximab maintenance did not demonstrate a statistically significant OS benefit in the phase 3 MAINTAIN trial, treating physicians should discuss the benefits and risks of maintenance therapy with their patient.

The same treatment options available in the first-line setting may be used upon relapse. Consideration should be made for retreatment with initial therapy if patients achieved a PFS beyond 2–4 years with this therapy. Bruton’s tyrosine kinase inhibitors are an important alternative to chemotherapy for all subtypes of relapsed MZL, with a preference growing for second-generation BTK inhibitors such as zanubrutinib given their improved toxicity profile. Zanubrutinib and ibrutinib are currently available through compassionate access across Canada, although the extent of future access is uncertain. 

Access to novel therapies remains challenging for MZL as the rarity of the disease and lack of adequate comparators make appropriately powered phase 3 randomized trials difficult to conduct. Regimens such as R2 would offer another tolerable, chemotherapy-free treatment option; however, R2 is not uniformly accessible for the treatment of MZL across all Canadian provinces. An unmet need also remains for the small subgroup of patients with poor responses to conventional treatments. Several novel CD19-directed therapies are being evaluated in clinical trials in patients with heavily treated, refractory B-cell malignancies, including tafasitimab in combination with lenalidomide, loncastuximab tesirine (an antibody-drug conjugate), blinatumomab (a bispecific T-cell engager), and axicabtagene ciloleucel or tisagenlecleucel (CAR T-cell therapies), which have demonstrated encouraging responses in patients with diffuse large B-cell lymphoma [98,99,100,101,102,103]. Given the lack of strong clinical evidence to support the optimal use of the majority of MZL treatments, clinical trial enrollment and well-designed real-world studies are needed to better understand therapy sequencing in MZL.

## Data Availability

Not applicable.

## References

[1] Sriskandarajah P., Dearden C.E. (2017). Epidemiology and environmental aspects of marginal zone lymphomas. Best Pract. Res. Clin. Haematol..

[2] Goede V. (2017). Marginal zone lymphoma in elderly and geriatric patients. Best Pract. Res. Clin. Haematol..

[3] Cerhan J.R., Habermann T.M. (2021). Epidemiology of marginal zone lymphoma. Ann. Lymphoma.

[4] Di Rocco A., Petrucci L., Assanto G.M., Martelli M., Pulsoni A. (2022). Extranodal Marginal Zone Lymphoma: Pathogenesis, Diagnosis and Treatment. Cancers.

[5] Cheah C.Y., Zucca E., Rossi D., Habermann T.M. (2022). Marginal zone lymphoma: Present status and future perspectives. Haematologica.

[6] Luminari S., Merli M., Rattotti S., Tarantino V., Marcheselli L., Cavallo F., Varettoni M., Bianchi B., Merli F., Tedeschi A. (2019). Early progression as a predictor of survival in marginal zone lymphomas: An analysis from the FIL-NF10 study. Blood.

[7] Conconi A., Martinelli G., Thiéblemont C., Ferreri A.J., Devizzi L., Peccatori F., Ponzoni M., Pedrinis E., Dell’Oro S., Pruneri G. (2003). Clinical activity of rituximab in extranodal marginal zone B-cell lymphoma of MALT type. Blood.

[8] Laurent C., Cook J.R., Yoshino T., Quintanilla-Martinez L., Jaffe E.S. (2023). Follicular lymphoma and marginal zone lymphoma: How many diseases?. Virchows Archiv..

[9] Jaffe E.S., Harris N.L., Stein H., Vardiman J.W. (2001). World Health Organization Classification of Tumours. Pathology and Genetics of Tumours of Haematopoietic and Lymphoid Tissues.

[10] Berger F., Traverse-Glehen A., Felman P., Callet-Bauchu E., Baseggio L., Gazzo S., Thieblemont C., French M., Magaud J.P., Salles G. (2005). Clinicopathologic features of Waldenström’s macroglobulinemia and marginal zone lymphoma: Are they distinct or the same entity?. Clin. Lymphoma.

[11] Treon S.P., Xu L., Yang G., Zhou Y., Liu X., Cao Y., Sheehy P., Manning R.J., Patterson C.J., Tripsas C. (2012). MYD88 L265P somatic mutation in Waldenström’s macroglobulinemia. N. Engl. J. Med..

[12] Raderer M., Wöhrer S., Streubel B., Troch M., Turetschek K., Jäger U., Skrabs C., Gaiger A., Drach J., Puespoek A. (2006). Assessment of Disease Dissemination in Gastric Compared With Extragastric Mucosa-Associated Lymphoid Tissue Lymphoma Using Extensive Staging: A Single-Center Experience. J. Clin. Oncol..

[13] Santos T.S.d., Tavares R.S., Farias D.L.C.d. (2017). Splenic marginal zone lymphoma: A literature review of diagnostic and therapeutic challenges. Rev. Bras. de Hematol. e Hemoter..

[14] Epperla N., Zhao Q., Shea L., Karmali R., Torka P., Seijung Oh T., Anampa-Guzman A., Jordan Bruno X., Umyarova E., Lindsey K. (2022). Impact of Monoclonal Protein at Diagnosis on Outcomes in Patients with Marginal Zone Lymphoma: A Multicenter Cohort Study. Blood.

[15] Traverse-Glehen A., Baseggio L., Salles G., Felman P., Berger F. (2011). Splenic marginal zone B-cell lymphoma: A distinct clinicopathological and molecular entity. Recent advances in ontogeny and classification. Curr. Opin. Oncol..

[16] Gebhart J., Lechner K., Skrabs C., Sliwa T., Müldür E., Ludwig H., Nösslinger T., Vanura K., Stamatopoulos K., Simonitsch-Klupp I. (2014). Lupus anticoagulant and thrombosis in splenic marginal zone lymphoma. Thromb. Res..

[17] Basset M., Defrancesco I., Milani P., Nuvolone M., Rattotti S., Foli A., Mangiacavalli S., Varettoni M., Benvenuti P., Cartia C.S. (2020). Nonlymphoplasmacytic lymphomas associated with light-chain amyloidosis. Blood.

[18] Stübgen J.-P. (2015). Autoantibody-Mediated Sensory Polyneuropathy Associated with Indolent B-Cell Non-Hodgkin’s Lymphoma: A Report of Two Cases. J. Clin. Neurol..

[19] Zucca E., Arcaini L., Buske C., Johnson P.W., Ponzoni M., Raderer M., Ricardi U., Salar A., Stamatopoulos K., Thieblemont C. (2020). Marginal zone lymphomas: ESMO Clinical Practice Guidelines for diagnosis, treatment and follow-up. Ann. Oncol..

[20] Van Den Brand M., Van Krieken J.H.J.M. (2013). Recognizing nodal marginal zone lymphoma: Recent advances and pitfalls. A systematic review. Haematologica.

[21] Perrone S., D’Elia G.M., Annechini G., Ferretti A., Tosti M.E., Foà R., Pulsoni A. (2016). Splenic marginal zone lymphoma: Prognostic factors, role of watch and wait policy, and other therapeutic approaches in the rituximab era. Leuk Res..

[22] Fischbach W., Dorlöchter C. (2019). Patients with gastric MALT lymphoma revealing persisting endoscopic abnormalities after successful eradication of Helicobacter pylori can be safely managed by a watch-and-wait strategy. Z. Gastroenterol..

[23] Joffe E., Leyfman Y., Drill E., Rajeeve S., Zelenetz A.D., Palomba M.L., Moskowitz C.H., Portlock C., Noy A., Horwitz S.M. (2021). Active surveillance of primary extranodal marginal zone lymphoma of bronchus-associated lymphoid tissue. Blood Adv..

[24] Lin H., Zhou K., Peng Z., Liang L., Cao J., Mei J. (2022). Surgery and chemotherapy cannot improve the survival of patients with early-stage mucosa-associated lymphoid tissue derived primary pulmonary lymphoma. Front. Oncol..

[25] Luminari S., Cesaretti M., Marcheselli L., Rashid I., Madrigali S., Maiorana A., Federico M. (2010). Decreasing incidence of gastric MALT lymphomas in the era of anti-Helicobacter pylori interventions: Results from a population-based study on extranodal marginal zone lymphomas. Ann. Oncol..

[26] Parsonnet J., Hansen S., Rodriguez L., Gelb A.B., Warnke R.A., Jellum E., Orentreich N., Vogelman J.H., Friedman G.D. (1994). Helicobacter pylori Infection and Gastric Lymphoma. N. Engl. J. Med..

[27] Zullo A., Hassan C., Andriani A., Cristofari F., De Francesco V., Ierardi E., Tomao S., Morini S., Vaira D. (2009). Eradication therapy for Helicobacter pylori in patients with gastric MALT lymphoma: A pooled data analysis. Am. J. Gastroenterol..

[28] Jung K., Kim D.H., Seo H.I., Gong E.J., Bang C.S. (2021). Efficacy of eradication therapy in Helicobacter pylori-negative gastric mucosa-associated lymphoid tissue lymphoma: A meta-analysis. Helicobacter.

[29] Xie Y.-L., He C.-Y., Wei S.-Q., Guan W.-J., Jiang Z. (2020). Clinical efficacy of the modified Helicobacter pylori eradication therapy for Helicobacter pylori-negative gastric mucosa-associated lymphoid tissue lymphoma: A meta analysis. Chin. Med. J..

[30] Liu H., Ye H., Ruskone-Fourmestraux A., De Jong D., Pileri S., Thiede C., Lavergne A., Boot H., Caletti G., Wündisch T. (2002). T(11;18) is a marker for all stage gastric MALT lymphomas that will not respond to H. pylori eradication. Gastroenterology.

[31] Ferreri A.J.M., Ponzoni M., Guidoboni M., Resti A.G., Politi L.S., Cortelazzo S., Demeter J., Zallio F., Palmas A., Muti G. (2006). Bacteria-Eradicating Therapy With Doxycycline in Ocular Adnexal MALT Lymphoma: A Multicenter Prospective Trial. JNCI J. Natl. Cancer Inst..

[32] Chanudet E., Zhou Y., Bacon C.M., Wotherspoon A.C., Müller-Hermelink H.K., Adam P., Dong H.Y., de Jong D., Li Y., Wei R. (2006). Chlamydia psittaci is variably associated with ocular adnexal MALT lymphoma in different geographical regions. J. Pathol..

[33] Vargas R.L., Fallone E., Felgar R.E., Friedberg J.W., Arbini A.A., Andersen A.A., Rothberg P.G. (2006). Is there an association between ocular adnexal lymphoma and infection with Chlamydia psittaci? The University of Rochester experience. Leuk Res..

[34] Aigelsreiter A., Leitner E., Deutsch A.J., Kessler H.H., Stelzl E., Beham-Schmid C., Beham A., Krugmann J., Dinges H.P., Linkesch W. (2008). Chlamydia psittaci in MALT lymphomas of ocular adnexals: The Austrian experience. Leuk Res..

[35] Collina F., Chiara A.D., Renzo A.D., Rosa G.D., Botti G., Franco R. (2012). Chlamydia psittaci in ocular adnexa MALT lymphoma: A possible role in lymphomagenesis and a different geographical distribution. Infect. Agents Cancer.

[36] Köller M.C., Aigelsreiter A. (2018). Chlamydia psittaci in Ocular Adnexal MALT Lymphoma: A Possible Causative Agent in the Pathogenesis of This Disease. Curr. Clin. Microbiol. Rep..

[37] Roggero E., Zucca E., Mainetti C., Bertoni F., Valsangiacomo C., Pedrinis E., Borisch B., Piffaretti J.C., Cavalli F., Isaacson P.G. (2000). Eradication of Borrelia burgdorferi infection in primary marginal zone B-cell lymphoma of the skin. Hum. Pathol..

[38] Kütting B., Bonsmann G., Metze D., Luger T.A., Cerroni L. (1997). Borrelia burgdorferi-associated primary cutaneous B cell lymphoma: Complete clearing of skin lesions after antibiotic pulse therapy or intralesional injection of interferon alfa-2a. J. Am. Acad. Dermatol..

[39] Lecuit M., Abachin E., Martin A., Poyart C., Pochart P., Suarez F., Bengoufa D., Feuillard J., Lavergne A., Gordon J.I. (2004). Immunoproliferative Small Intestinal Disease Associated with Campylobacter jejuni. N. Engl. J. Med..

[40] Kassan S.S., Thomas T.L., Moutsopoulos H.M., Hoover R., Kimberly R.P., Budman D.R., Costa J., Decker J.L., Chused T.M. (1978). Increased risk of lymphoma in sicca syndrome. Ann. Intern. Med..

[41] Royer B., Cazals-Hatem D., Sibilia J., Agbalika F., Cayuela J.-M., Soussi T., Maloisel F.D.R., Clauvel J.-P., Brouet J.-C., Mariette X. (1997). Lymphomas in Patients With Sjögren’s Syndrome Are Marginal Zone B-Cell Neoplasms, Arise in Diverse Extranodal and Nodal Sites, and Are Not Associated With Viruses. Blood.

[42] Voulgarelis M., Dafni U.G., Isenberg D.A., Moutsopoulos H.M. (1999). Malignant lymphoma in primary Sjögren’s syndrome: A multicenter, retrospective, clinical study by the European concerted action on Sjögren’s syndrome. Arthritis Rheum..

[43] Derringer G.A., Thompson L.D., Frommelt R.A., Bijwaard K.E., Heffess C.S., Abbondanzo S.L. (2000). Malignant lymphoma of the thyroid gland: A clinicopathologic study of 108 cases. Am. J. Surg. Pathol..

[44] Karvounis E., Kappas I., Angelousi A., Makris G.-M., Kassi E. (2020). Mucosa-Associated Lymphoid Tissue Lymphoma of the Thyroid Gland: A Systematic Review of the Literature. Eur. Thyroid J..

[45] Mele A., Pulsoni A., Bianco E., Musto P., Szklo A., Sanpaolo M.G., Iannitto E., De Renzo A., Martino B., Liso V. (2003). Hepatitis C virus and B-cell non-Hodgkin lymphomas: An Italian multicenter case-control study. Blood.

[46] Kelaidi C., Rollot F., Park S., Tulliez M., Christoforov B., Calmus Y., Podevin P., Bouscary D., Sogni P., Blanche P. (2004). Response to antiviral treatment in hepatitis C virus-associated marginal zone lymphomas. Leukemia.

[47] Arcaini L., Vallisa D., Rattotti S., Ferretti V.V., Ferreri A.J.M., Bernuzzi P., Merli M., Varettoni M., Chiappella A., Ambrosetti A. (2014). Antiviral treatment in patients with indolent B-cell lymphomas associated with HCV infection: A study of the Fondazione Italiana Linfomi. Ann. Oncol..

[48] Yahalom J., Xu A.J., Noy A., Lobaugh S., Chelius M., Chau K., Portlock C., Hajj C., Imber B.S., Straus D.J. (2021). Involved-site radiotherapy for Helicobacter pylori–independent gastric MALT lymphoma: 26 years of experience with 178 patients. Blood Adv..

[49] Goda J.S., Gospodarowicz M., Pintilie M., Wells W., Hodgson D.C., Sun A., Crump M., Tsang R.W. (2010). Long-term outcome in localized extranodal mucosa-associated lymphoid tissue lymphomas treated with radiotherapy. Cancer.

[50] Lowry L., Smith P., Qian W., Falk S., Benstead K., Illidge T., Linch D., Robinson M., Jack A., Hoskin P. (2011). Reduced dose radiotherapy for local control in non-Hodgkin lymphoma: A randomised phase III trial. Radiother. Oncol..

[51] Yahalom J., Illidge T., Specht L., Hoppe R.T., Li Y.-X., Tsang R., Wirth A. (2015). Modern Radiation Therapy for Extranodal Lymphomas: Field and Dose Guidelines From the International Lymphoma Radiation Oncology Group. Int. J. Radiat. Oncol. Biol. Phys..

[52] Pinnix C.C., Dabaja B.S., Milgrom S.A., Smith G.L., Abou Z., Nastoupil L., Romaguera J., Turturro F., Fowler N., Fayad L. (2017). Ultra-low-dose radiotherapy for definitive management of ocular adnexal B-cell lymphoma. Head Neck.

[53] Cerrato M., Orlandi E., Vella A., Bartoncini S., Iorio G.C., Bongiovanni D., Capriotti F., Boccomini C., Vassallo F., Cavallin C. (2021). Efficacy of low-dose radiotherapy (2 Gy × 2) in the treatment of marginal zone and mucosa-associated lymphoid tissue lymphomas. Br. J. Radiol..

[54] ChacóN J.I., Mollejo M., MuñOz E., Algara P., Mateo M., Lopez L., Andrade J.S., Carbonero I.G.A., MartıίNez B., Piris M.A. (2002). Splenic marginal zone lymphoma: Clinical characteristics and prognostic factors in a series of 60 patients. Blood.

[55] Xing K.H., Kahlon A., Skinnider B.F., Connors J.M., Gascoyne R.D., Sehn L.H., Savage K.J., Slack G.W., Shenkier T.N., Klasa R. (2015). Outcomes in splenic marginal zone lymphoma: Analysis of 107 patients treated in British Columbia. Br. J. Haematol..

[56] Kalpadakis C., Pangalis G.A., Angelopoulou M.K., Sachanas S., Kontopidou F.N., Yiakoumis X., Kokoris S.I., Dimitriadou E.M., Dimopoulou M.N., Moschogiannis M. (2013). Treatment of Splenic Marginal Zone Lymphoma With Rituximab Monotherapy: Progress Report and Comparison With Splenectomy. Oncologist.

[57] Lenglet J., Traullé C., Mounier N., Benet C., Munoz-Bongrand N., Amorin S., Noguera M.E., Traverse-Glehen A., Ffrench M., Baseggio L. (2014). Long-term follow-up analysis of 100 patients with splenic marginal zone lymphoma treated with splenectomy as first-line treatment. Leuk Lymphoma.

[58] Pata G., Damiani E., Bartoli M., Solari S., Anastasia A., Pagani C., Tucci A., Ragni F. (2016). Peri-operative complications and hematologic improvement after first-line splenectomy for splenic marginal zone lymphoma. Leuk. Lymphoma.

[59] Arcaini L., Rossi D., Paulli M. (2016). Splenic marginal zone lymphoma: From genetics to management. Blood.

[60] Lossos I.S., Morgensztern D., Blaya M., Alencar A., Pereira D., Rosenblatt J. (2007). Rituximab for treatment of chemoimmunotherapy naive marginal zone lymphoma. Leuk. Lymphoma.

[61] Raderer M., Jäger G., Brugger S., Püspök A., Fiebiger W., Drach J., Wotherspoon A., Chott A. (2003). Rituximab for treatment of advanced extranodal marginal zone B cell lymphoma of the mucosa-associated lymphoid tissue lymphoma. Oncology.

[62] Tsimberidou A.M., Catovsky D., Schlette E., O’Brien S., Wierda W.G., Kantarjian H., Garcia-Manero G., Wen S., Do K.-A., Lerner S. (2006). Outcomes in patients with splenic marginal zone lymphoma and marginal zone lymphoma treated with rituximab with or without chemotherapy or chemotherapy alone. Cancer.

[63] Bennett M., Sharma K., Yegena S., Gavish I., Dave H.P., Schechter G.P. (2005). Rituximab monotherapy for splenic marginal zone lymphoma. Haematologica.

[64] Kalpadakis C., Pangalis G.A., Sachanas S., Tsirkinidis P., Kontopidou F.N., Moschogiannis M., Yiakoumis X., Koulieris E., Dimopoulou M.N., Kokkoris S.I. (2018). Rituximab monotherapy in splenic marginal zone lymphoma: Prolonged responses and potential benefit from maintenance. Blood.

[65] Flinn I.W., Van Der Jagt R., Kahl B.S., Wood P., Hawkins T.E., Macdonald D., Hertzberg M., Kwan Y.-L., Simpson D., Craig M. (2014). Randomized trial of bendamustine-rituximab or R-CHOP/R-CVP in first-line treatment of indolent NHL or MCL: The BRIGHT study. Blood.

[66] Rummel M.J., Niederle N., Maschmeyer G., Banat G.A., von Grünhagen U., Losem C., Kofahl-Krause D., Heil G., Welslau M., Balser C. (2013). Bendamustine plus rituximab versus CHOP plus rituximab as first-line treatment for patients with indolent and mantle-cell lymphomas: An open-label, multicentre, randomised, phase 3 non-inferiority trial. Lancet.

[67] Rummel M.J., Maschmeyer G., Ganser A., Heider A., von Gruenhagen U., Losem C., Heil G., Welslau M., Balser C., Kaiser U. (2017). Bendamustine plus rituximab (B-R) versus CHOP plus rituximab (CHOP-R) as first-line treatment in patients with indolent lymphomas: Nine-year updated results from the StiL NHL1 study. J. Clin. Oncol..

[68] Salar A., Domingo-Domenech E., Panizo C., Nicolás C., Bargay J., Muntañola A., Canales M., Bello J.L., Sancho J.M., Tomás J.F. (2017). Long-term results of a phase 2 study of rituximab and bendamustine for mucosa-associated lymphoid tissue lymphoma. Blood.

[69] Iannitto E., Bellei M., Amorim S., Ferreri A.J.M., Marcheselli L., Cesaretti M., Haioun C., Mancuso S., Bouabdallah K., Gressin R. (2018). Efficacy of bendamustine and rituximab in splenic marginal zone lymphoma: Results from the phase II BRISMA/IELSG36 study. Br. J. Haematol..

[70] Zucca E., Conconi A., Martinelli G., Bouabdallah R., Tucci A., Vitolo U., Martelli M., Pettengell R., Salles G., Sebban C. (2017). Final Results of the IELSG-19 Randomized Trial of Mucosa-Associated Lymphoid Tissue Lymphoma: Improved Event-Free and Progression-Free Survival With Rituximab Plus Chlorambucil Versus Either Chlorambucil or Rituximab Monotherapy. J. Clin. Oncol..

[71] Salar A., Domingo-Domenech E., Estany C., Canales M.A., Gallardo F., Servitje O., Fraile G., Montalbán C. (2009). Combination therapy with rituximab and intravenous or oral fludarabine in the first-line, systemic treatment of patients with extranodal marginal zone B-cell lymphoma of the mucosa-associated lymphoid tissue type. Cancer.

[72] Brown J.R., Friedberg J.W., Feng Y., Scofield S., Phillips K., Dal Cin P., Joyce R., Takvorian R.W., Fisher D.C., Fisher R.I. (2009). A phase 2 study of concurrent fludarabine and rituximab for the treatment of marginal zone lymphomas. Br. J. Haematol..

[73] Rummel M.J., Koenigsmann M., Chow K.U., Knauf W., Lerchenmuller C.A., Losem C., Goerner M., Hertenstein B., Decker T., Ganser A. (2018). Two years rituximab maintenance vs. observation after first line treatment with bendamustine plus rituximab (B-R) in patients with marginal zone lymphoma (MZL): Results of a prospective, randomized, multicenter phase 2 study (the StiL NHL7-2008 MAINTAIN trial). J. Clin. Oncol..

[74] Aksoy S., Dizdar O., Hayran M., Harputluoğlu H. (2009). Infectious complications of rituximab in patients with lymphoma during maintenance therapy: A systematic review and meta-analysis. Leuk Lymphoma.

[75] Shree T., Shankar V., Lohmeyer J.J.K., Czerwinski D.K., Schroers-Martin J.G., Rodriguez G.M., Beygi S., Kanegai A.M., Corbelli K.S., Gabriel E. (2022). CD20-Targeted Therapy Ablates De Novo Antibody Response to Vaccination but Spares Preestablished Immunity. Blood Cancer Discov..

[76] Vijenthira A., Gong I., Betschel S.D., Cheung M., Hicks L.K. (2021). Vaccine response following anti-CD20 therapy: A systematic review and meta-analysis of 905 patients. Blood Adv..

[77] Fowler N.H., Davis R.E., Rawal S., Nastoupil L., Hagemeister F.B., McLaughlin P., Kwak L.W., Romaguera J.E., Fanale M.A., Fayad L.E. (2014). Safety and activity of lenalidomide and rituximab in untreated indolent lymphoma: An open-label, phase 2 trial. Lancet Oncol..

[78] Kiesewetter B., Willenbacher E., Willenbacher W., Egle A., Neumeister P., Voskova D., Mayerhoefer M.E., Simonitsch-Klupp I., Melchardt T., Greil R. (2017). A phase 2 study of rituximab plus lenalidomide for mucosa-associated lymphoid tissue lymphoma. Blood.

[79] Flinn I.W., Van Der Jagt R., Kahl B., Wood P., Hawkins T., Macdonald D., Simpson D., Kolibaba K., Issa S., Chang J. (2019). First-Line Treatment of Patients With Indolent Non-Hodgkin Lymphoma or Mantle-Cell Lymphoma With Bendamustine Plus Rituximab Versus R-CHOP or R-CVP: Results of the BRIGHT 5-Year Follow-Up Study. J. Clin. Oncol..

[80] Salar A., Domingo-Domenech E., Panizo C., Nicolás C., Bargay J., Muntañola A., Canales M., Bello J.L., Sancho J.M., Tomás J.F. (2014). First-line response-adapted treatment with the combination of bendamustine and rituximab in patients with mucosa-associated lymphoid tissue lymphoma (MALT2008-01): A multicentre, single-arm, phase 2 trial. Lancet Haematol..

[81] Dreyling M., Santoro A., Mollica L., Leppä S., Follows G., Lenz G., Kim W.S., Nagler A., Dimou M., Demeter J. (2020). Long-term safety and efficacy of the PI3K inhibitor copanlisib in patients with relapsed or refractory indolent lymphoma: 2-year follow-up of the CHRONOS-1 study. Am. J. Hematol..

[82] United States Food & Drug Administration FDA Investigating Possible Increased Risk of Death with Lymphoma Medicine Ukoniq (umbralisib). https://www.fda.gov/drugs/development-approval-process-drugs/fda-investigating-possible-increased-risk-death-lymphoma-medicine-ukoniq-umbralisib.

[83] Gopal A.K., Kahl B.S., De Vos S., Wagner-Johnston N.D., Schuster S.J., Jurczak W.J., Flinn I.W., Flowers C.R., Martin P., Viardot A. (2014). PI3Kδ Inhibition by Idelalisib in Patients with Relapsed Indolent Lymphoma. N. Engl. J. Med..

[84] Flinn I.W., Miller C.B., Ardeshna K.M., Tetreault S., Assouline S.E., Mayer J., Merli M., Lunin S.D., Pettitt A.R., Nagy Z. (2019). DYNAMO: A Phase II Study of Duvelisib (IPI-145) in Patients With Refractory Indolent Non-Hodgkin Lymphoma. J. Clin. Oncol..

[85] Dreyling M., Santoro A., Mollica L., Leppä S., Follows G.A., Lenz G., Kim W.S., Nagler A., Panayiotidis P., Demeter J. (2017). Phosphatidylinositol 3-Kinase Inhibition by Copanlisib in Relapsed or Refractory Indolent Lymphoma. J. Clin. Oncol..

[86] Matasar M.J., Capra M., Özcan M., Lv F., Li W., Yañez E., Sapunarova K., Lin T., Jin J., Jurczak W. (2021). Copanlisib plus rituximab versus placebo plus rituximab in patients with relapsed indolent non-Hodgkin lymphoma (CHRONOS-3): A double-blind, randomised, placebo-controlled, phase 3 trial. Lancet Oncol..

[87] Freeman C.L., Kridel R., Moccia A.A., Savage K.J., Villa D.R., Scott D.W., Gerrie A.S., Ferguson D., Cafferty F., Slack G.W. (2019). Early progression after bendamustine-rituximab is associated with high risk of transformation in advanced stage follicular lymphoma. Blood.

[88] Salles G.A., Morschhauser F., Solal-Céligny P., Thieblemont C., Lamy T., Tilly H., Gyan E., Lei G., Wenger M., Wassner-Fritsch E. (2013). Obinutuzumab (GA101) in Patients With Relapsed/Refractory Indolent Non-Hodgkin Lymphoma: Results from the Phase II GAUGUIN Study. J. Clin. Oncol..

[89] Sehn L.H., Chua N., Mayer J., Dueck G., Trněný M., Bouabdallah K., Fowler N., Delwail V., Press O., Salles G. (2016). Obinutuzumab plus bendamustine versus bendamustine monotherapy in patients with rituximab-refractory indolent non-Hodgkin lymphoma (GADOLIN): A randomised, controlled, open-label, multicentre, phase 3 trial. Lancet Oncol..

[90] Noy A., De Vos S., Coleman M., Martin P., Flowers C.R., Thieblemont C., Morschhauser F., Collins G.P., Ma S., Peles S. (2020). Durable ibrutinib responses in relapsed/refractory marginal zone lymphoma: Long-term follow-up and biomarker analysis. Blood Adv..

[91] Opat S., Tedeschi A., Linton K., McKay P., Hu B., Chan H., Jin J., Sobieraj-Teague M., Zinzani P.L., Coleman M. (2021). The MAGNOLIA Trial: Zanubrutinib, a Next-Generation Bruton Tyrosine Kinase Inhibitor, Demonstrates Safety and Efficacy in Relapsed/Refractory Marginal Zone Lymphoma. Clin. Cancer Res..

[92] Opat S., Tedeschi A., Hu B., Linton K.M., McKay P., Chan H., Jin J., Sun M., Sobieraj-Teague M., Zinzani P.L. (2022). Long-Term Efficacy and Safety of Zanubrutinib in Patients with Relapsed/Refractory (R/R) Marginal Zone Lymphoma (MZL): Final Analysis of the Magnolia (BGB-3111-214) Trial. Blood.

[93] Tam C.S., Opat S., D’Sa S., Jurczak W., Lee H.-P., Cull G., Owen R.G., Marlton P., Wahlin B.E., Sanz R.G. (2020). A randomized phase 3 trial of zanubrutinib vs ibrutinib in symptomatic Waldenström macroglobulinemia: The ASPEN study. Blood.

[94] Brown J.R., Eichhorst B., Hillmen P., Jurczak W., Kaźmierczak M., Lamanna N., O’Brien S.M., Tam C.S., Qiu L., Zhou K. (2022). Zanubrutinib or Ibrutinib in Relapsed or Refractory Chronic Lymphocytic Leukemia. N. Engl. J. Med..

[95] Leonard J.P., Trneny M., Izutsu K., Fowler N.H., Hong X., Zhu J., Zhang H., Offner F., Scheliga A., Nowakowski G.S. (2019). AUGMENT: A Phase III Study of Lenalidomide Plus Rituximab Versus Placebo Plus Rituximab in Relapsed or Refractory Indolent Lymphoma. J. Clin. Oncol..

[96] Kiesewetter B., Greil R., Willenbacher W., Neumeister P., Fridrik M.A., Markus R. (2015). AGMT MALT-2: A Phase II Study of Rituximab Plus Lenalidomide in Patients with Extranodal Marginal Zone B-Cell Lymphoma of the Mucosa-Associated Lymphoid Tissue (MALT lymphoma). Blood.

[97] Fowler N.H., Samaniego F., Jurczak W., Ghosh N., Derenzini E., Reeves J.A., Knopińska-Posłuszny W., Cheah C.Y., Phillips T., Lech-Maranda E. (2021). Umbralisib, a Dual PI3Kδ/CK1ε Inhibitor in Patients With Relapsed or Refractory Indolent Lymphoma. J. Clin. Oncol..

[98] Zinzani P.L., Minotti G. (2022). Anti-CD19 monoclonal antibodies for the treatment of relapsed or refractory B-cell malignancies: A narrative review with focus on diffuse large B-cell lymphoma. J. Cancer Res. Clin. Oncol..

[99] Neelapu S.S., Locke F.L., Bartlett N.L., Lekakis L.J., Miklos D.B., Jacobson C.A., Braunschweig I., Oluwole O.O., Siddiqi T., Lin Y. (2017). Axicabtagene Ciloleucel CAR T-Cell Therapy in Refractory Large B-Cell Lymphoma. N. Engl. J. Med..

[100] Schuster S.J., Bishop M.R., Tam C.S., Waller E.K., Borchmann P., McGuirk J.P., Jäger U., Jaglowski S., Andreadis C., Westin J.R. (2019). Tisagenlecleucel in Adult Relapsed or Refractory Diffuse Large B-Cell Lymphoma. N. Engl. J. Med..

[101] Salles G., Duell J., González Barca E., Tournilhac O., Jurczak W., Liberati A.M., Nagy Z., Obr A., Gaidano G., André M. (2020). Tafasitamab plus lenalidomide in relapsed or refractory diffuse large B-cell lymphoma (L-MIND): A multicentre, prospective, single-arm, phase 2 study. Lancet Oncol..

[102] Caimi P.F., Ai W., Alderuccio J.P., Ardeshna K.M., Hamadani M., Hess B., Kahl B.S., Radford J., Solh M., Stathis A. (2021). Loncastuximab tesirine in relapsed or refractory diffuse large B-cell lymphoma (LOTIS-2): A multicentre, open-label, single-arm, phase 2 trial. Lancet Oncol..

[103] Viardot A., Goebeler M.-E., Hess G., Neumann S., Pfreundschuh M., Adrian N., Zettl F., Libicher M., Sayehli C., Stieglmaier J. (2016). Phase 2 study of the bispecific T-cell engager (BiTE) antibody blinatumomab in relapsed/refractory diffuse large B-cell lymphoma. Blood.

[104] Brice P., Bastion Y., Lepage E., Brousse N., Haïoun C., Moreau P., Straetmans N., Tilly H., Tabah I., Solal-Céligny P. (1997). Comparison in low-tumor-burden follicular lymphomas between an initial no-treatment policy, prednimustine, or interferon alfa: A randomized study from the Groupe d’Etude des Lymphomes Folliculaires. Groupe d’Etude des Lymphomes de l’Adulte. J. Clin. Oncol..

[105] Wilder R.B., Jones D., Tucker S.L., Fuller L.M., Ha C.S., McLaughlin P., Hess M.A., Cabanillas F., Cox J.D. (2001). Long-term results with radiotherapy for Stage I-II follicular lymphomas. Int. J. Radiat. Oncol. Biol. Phys..

[106] Petersen P.M., Gospodarowicz M., Tsang R., Pintilie M., Wells W., Hodgson D., Sun A., Crump M., Patterson B., Bailey D. (2004). Long-term outcome in stage I and II follicular lymphoma following treatment with involved field radiation therapy alone. J. Clin. Oncol..

